# Multilevel Parallelization of AutoDock 4.2

**DOI:** 10.1186/1758-2946-3-12

**Published:** 2011-04-28

**Authors:** Andrew P Norgan, Paul K Coffman, Jean-Pierre A Kocher, David J Katzmann, Carlos P Sosa

**Affiliations:** 1 Department of Biochemistry and Molecular Biology, Mayo Clinic, Rochester, MN, USA; 2 IBM, Rochester, MN, USA; 3 Division of Biomedical Statistics & Informatics, Mayo Clinic, Rochester, MN, USA; 4 Biomedical Informatics and Computational Biology Program, University of Minnesota, Rochester, MN, USA

## Abstract

**Background:**

Virtual (computational) screening is an increasingly important tool for drug discovery. AutoDock is a popular open-source application for performing molecular docking, the prediction of ligand-receptor interactions. AutoDock is a serial application, though several previous efforts have parallelized various aspects of the program. In this paper, we report on a multi-level parallelization of AutoDock 4.2 (mpAD4).

**Results:**

Using MPI and OpenMP, AutoDock 4.2 was parallelized for use on MPI-enabled systems and to multithread the execution of individual docking jobs. In addition, code was implemented to reduce input/output (I/O) traffic by reusing grid maps at each node from docking to docking. Performance of mpAD4 was examined on two multiprocessor computers.

**Conclusions:**

Using MPI with OpenMP multithreading, mpAD4 scales with near linearity on the multiprocessor systems tested. In situations where I/O is limiting, reuse of grid maps reduces both system I/O and overall screening time. Multithreading of AutoDock's Lamarkian Genetic Algorithm with OpenMP increases the speed of execution of individual docking jobs, and when combined with MPI parallelization can significantly reduce the execution time of virtual screens. This work is significant in that mpAD4 speeds the execution of certain molecular docking workloads and allows the user to optimize the degree of system-level (MPI) and node-level (OpenMP) parallelization to best fit both workloads and computational resources.

## Background

Virtual screening, the use of computers to predict the binding of libraries of small molecules to known target structures, is an increasingly important component of the drug discovery process [1,2]. Although high-throughput biochemical screening is still the predominant technique for lead compound discovery, the success of *in silico *screening in identifying drug leads has led to the growing use of virtual screening as a complement to traditional empirical methods [3,4]. There are a large number of software packages for conducting the molecular docking simulations used in virtual screening, with the open-source packages AutoDock and DOCK, and the commercial packages GOLD, FlexX and ICM, among the most popular [5]. Of those five packages the most widely cited is AutoDock, which has been successfully used in a number of virtual screens and in the development of the HIV integrase inhibitor raltegravir [5-7]. This work is focused on AutoDock's most recent major version, AutoDock 4.2 [8].

In its current iteration, AutoDock 4.2's (AD4) default search function is a Lamarkian Genetic Algorithm (LGA), a hybrid genetic algorithm with local optimization that uses a parameterized free-energy scoring function to estimate binding energy [8,9]. To perform a ligand-receptor docking experiment, the software accepts as inputs ligand and macromolecule coordinates, and then utilizes the LGA to generate ligand positions and minimize binding energies using precalculated pairwise potential grid maps [10]. Each *docking *is comprised of multiple independent executions of the LGA, limited to a user specified number of energy evaluations (ga_evals) or generations (ga_num_generations). The individual LGA executions (ga_runs) are clustered and ranked to generate the final docking result.

While AD4 has been widely used for virtual screening, one limitation to its usefulness is its docking speed [11,12]. A potential way to increase AD4 performance is to parallelize aspects of its execution. Trends in processor architecture (multicore and multithreaded), and the increasing importance of highly parallel hardware such as graphics cards in scientific computation, underscore the importance of optimizing applications for parallel workloads. AD4 is a serial application not originally designed for computational clusters or to take advantage of parallel processing. There have been several previous efforts to parallelize aspects of AD4 and enable its use on high performance clusters, including: DOVIS and DOVIS 2.0 (Linux/UNIX clusters), Dockres (Linux/UNIX clusters), VSDocker (Windows clusters), and recently Autodock4.lga.MPI (an MPI implementation of Autodock4) [13-17]. In general, these programs either encapsulate AutoDock in code wrappers or supply scripts that automate aspects of the preparation, distribution, execution and load balancing of AutoDock on clusters. DOVIS 2.0 uses multithreading or SSH for cluster execution, while VSDocker utilizes MPICH2 or MSMPI for cluster communication [14,16]. Dockres runs in conjunction with several different cluster queuing systems, as does DOVIS 2.0 [15,16]. One challenge in parallelizing AutoDock for a cluster environment is that the program can generate significant network I/O during the loading of grid maps at the beginning of each docking, and when writing log files as dockings finish. Though log file writing can not easily be avoided, reuse of grid maps is a possibility as the majority of grid maps will be the same in each docking. One potential solution, if sufficient RAM is available, is to keep the grid maps in memory. This approach was used in both DOVIS and Autodock4.lga.MPI (with maps repackaged into an efficient binary format), with significant decreases in I/O observed when grid maps are loaded only once for each node [13,17].

In addition to optimizing AutoDock's execution on clusters, several previous efforts parallelized individual dockings. In a standard docking, the most time intensive task is the repeated execution of AutoDock's LGA, which is run tens or hundreds of times with identical structure files, grid maps and parameters. The LGA was the focus of parallelization efforts by Thormann and Pons, who parallelized the LGA of AutoDock 3.0 using OpenMP, and Khodade *et al*., who parallelized AutoDock 3.0 and a beta version of AutoDock 4.0 using MPI [18,19]. These approaches both resulted in a significant increase in AD4 execution speed, with Thormann and Pons reporting an approximately 95% × *N*(where N = 8) speedup, and Khodade *et al*. observing near linear speed increases on a 96-core POWER5 system [18,19].

Extending on these previous approaches, we had three goals for parallelization of AD4: 1) enable parallel execution of AD4 across multiple HPC architectures, 2) reduce I/O, and 3) parallelize the execution of individual docking jobs. Accordingly, we parallelized AD4 at multiple levels by: 1) utilizing MPI to distribute AD4 docking jobs across a system, 2) developing a grid map reuse scheme (conceptually similar to that implemented in DOVIS) to reduce I/O, and 3) implementing OpenMP parallelization of the LGA to achieve node-level parallelization. This standards-based parallelization scheme is significant in that it results in a highly portable parallel implementation of AD4 with user customizability in the balance between system-level and node-level parallel execution.

## Implementation

AutoDock 4.2 (AD4) was parallelized at multiple levels using the MPICH2 implementation of the MPI standard and OpenMP application programming interface, resulting in the parallel code mpAutoDock 4.2 (mpAD4). The implementation of MPI and OpenMP in mpAD4 is standards compliant and portable to any architecture with a suitable compiler. MPI was used to parallelize the main() function of AD4 to facilitate virtual screening on MPI-enabled clusters, while OpenMP was used to implement multi-threading of the AD4 LGA. Scaling of the mpAD4 code in multithreaded and serial operation was evaluated using an IBM BlueGene/P system and a 32-core IBM POWER7 server.

### MPI Parallelization

To facilitate system-level parallelization, the mpAD4 main() function was rewritten as a function call from the MPI driver. In this context, mpAD4 is executed within a master-slave scheme in which node-0 is the master node and all other nodes are slave nodes. The master node coordinates all docking activities by reading a list of docking directories from an ASCII file and then assigns individual dockings to specific slave nodes via MPI_Send(). Once the docking assignment has been received via MPI_Recv(), the slave nodes perform the docking work by loading necessary files, calling the mpAD4 main() function to dock the ligand that the master node has assigned to it, and writing the docking log file. To allow the user to monitor progress, the master writes three log files to track submitted dockings (MPI_Send() call from the master), successful dockings (MPI Send() call from a slave with data indicating docking success received by the master via MPI_Recv()) and failed dockings (MPI Send() call from a slave with data indicating docking failure received by the master via MPI_Recv()).

### I/O Optimization

**Figure 1:**
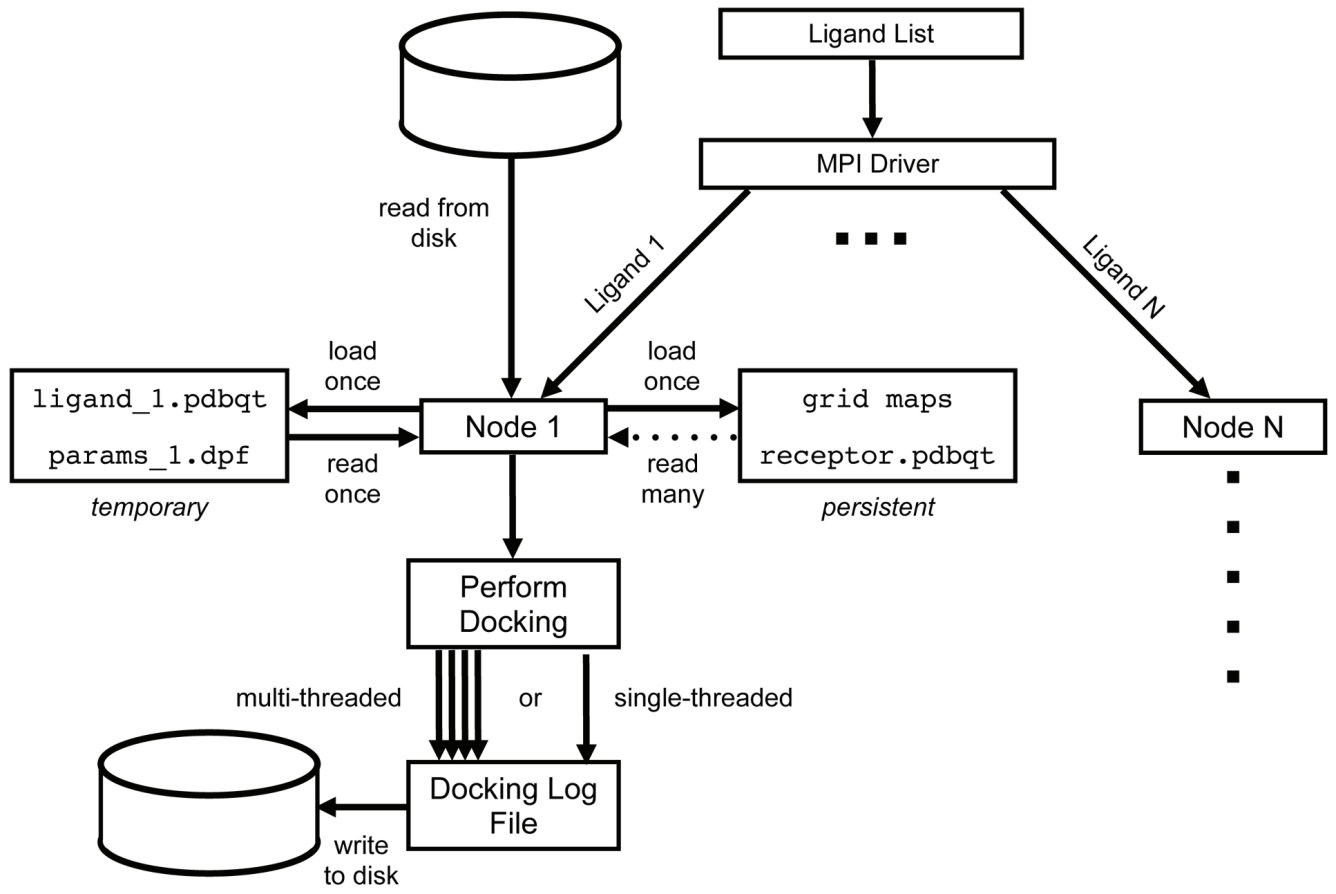
**Workflow of mpAutoDock**. mpAD4 uses an MPI-driver to distribute work to individual nodes. With grid map reuse enabled, the precomputed grid maps, receptor and docking parameter files are loaded at the node level and reused for each additional docking. An individual docking on each node can be parallelized by running multiple instances of Lamarkian Genetic Algorithm in parallel using OpenMP threads.

AD4 requires the precalculation of one electrostatic map, one desolvation map, and individual atomic affinity grid maps for each AD4 atom type found in the ligand(s). The default AD4 behavior is to load all grid maps required for a specific docking into memory from the file system and to release that memory at the end of the docking. Thus, when the next docking begins many of the same grid maps are reloaded. In addition to the time required to load the grid maps, this behavior generates significant I/O that is unnecessary given that different dockings utilize the same electrostatic and desolvation maps, and often atomic affinity maps. Therefore, a parameter has been added to the mpAD4 executable to control grid maps persistence from one docking to another on the slave nodes. With mpAD4, as the main() function begins execution on the slave the default behavior is to load all grid maps required to dock the first ligand into compute node memory. Any remaining atomic affinity maps are loaded for subsequent dockings at the node only when required by a ligand with a previously unused atom type. Once loaded, a map persists in node memory until program termination (Figure 1). To accomplish this, the scope of the multi-dimensional array holding the grid map data changed from local (in the main() function) to global, allowing the grid map data to persist from docking to docking on a slave node. In addition, the code that manages and references this grid map array was modified to initially load only atomic affinity maps required for the first docking, and then subsequently load appropriate atomic affinity maps when required. This approach minimizes startup I/O by loading the smallest possible amount of initial data onto the compute nodes. The user can specify grid map persistence at runtime using the flags (reload_maps or reuse_maps). Except were otherwise indicated, benchmarks were run with grid map reuse (gm = reuse).

### OpenMP Parallelization

The majority of an AD4 docking is spent within the search and scoring routines, making them appealing targets for parallelization. AD4 includes several search functions, including simulated annealing (SA), genetic algorithm (GA), local search (LS) and a hybrid GA/LS (LGA). The LGA was chosen for parallelization as it was previously demonstrated to outperform either the SA or GA alone, and the LS is useful primarily for minimizing already docked structures [10,18]. To parallelize the LGA with OpenMP, modifications to the input seed value generation and docking output handling code were required. The AD4 random number generator (RNG) utilizes a deterministic IGNLGI algorithm to generate a time-based random number seed for each LGA run. Thus, when OpenMP threads were created simultaneously with an unmodified RNG, each thread would receive an identical seed value. Therefore, the mpAD4 RNG was changed to include thread ID in the time-based seed passed to the RNG to generate unique seeds for each thread. The other code change required was related to how log information about each iteration of the LGA is written to the docking log. In the AD4, the LGA writes information to the docking log piecewise for each iteration. When the code was multithreaded, log information appeared scrambled as different threads simultaneously wrote LGA outputs. To resolve this issue in mpAD4, LGA outputs are buffered and then written en bloc after thread completion, thereby keeping the output of each ga_run contiguous within the docking log.

### Performance Profiling

In addition to the parallelization code, performance profiling has been added to mpAD4. Profiling can be turned on or off at compile time with a compiler directive. When profiling is enabled, a .csv file is updated as each docking finishes, so a user can monitor the progress of individual docking jobs and be made aware of any performance issues while the program is running. The profiling records calculation and communication start/stop times and durations from the moment the master sends the MPI message to the slave to the moment the master receives the return message from the slave with the docking status, and writes the values to a single comma delimited entry in the profiling log. Profiling outputs may be of interest to users of mpAD4 for characterizing performance bottlenecks on their system and for future developers of mpAD4. When not otherwise indicated, benchmarks were run with profiling enabled.

### Blue Gene/P and POWER7 architectures

In this study two architectures were used to test mpAD4 performance, an IBM Blue Gene/P (BG/P) system and a shared-memory 32-core POWER7 p755 server [20,21]. The BG/P system is composed of dense racks of IBM PowerPC 450 processors running at 850 MHz with 4 cores and 4 GB RAM per compute node connected by a high performance interconnect to a storage array running the General Parallel File System (GPFS). BG/P can be configured in several different modes, including symmetric multi-processing (SMP) and virtual node (VN) [21]. In SMP mode, each compute node executes a single task with a maximum of four threads, with node resources including memory and network bandwidth shared by all processes. In VN mode, four single-threaded tasks are run on each node, one task per core, with each task having access to 1/4 of the total node memory. Thus, in comparing VN and SMP mode, VN mode will run four times the number of simultaneous independent MPI tasks as SMP mode, but the same number of total CPU cores will are utilized in each mode. The SMP and VN modes were used to examine differences in mpAD4 scaling and performance using MPI with multithreading SMP(OMP = 4) or MPI alone VN(OMP = 1). BG/P compute nodes do not have local disk storage, and I/O requests to the storage array are handled by dedicated I/O nodes that communicate with the network file system. Compute nodes connect to I/O nodes via a high-bandwidth "global collective network" that moves process and application data to and from the I/O nodes [21]. Each compute and I/O Node has three bidirectional links to the global collective network at 850 MBps per link, for a total of 5.1 GBps bandwidth per node. I/O nodes, in turn, are connected to the external file filesystem by a 10 Gb ethernet link. A BG/P system can be configured to run with a variable number of I/O nodes to model I/O replete or I/O poor systems.

In this study we tested two configurations, I/O poor (1 I/O node per 512 CPU cores) and I/O replete (1 I/O node per 64 CPU cores). When not otherwise indicated, an I/O replete configuration was used. The p755 system is a POWER7 3.3 GHz server with 32 cores and 128 GB of RAM, running the AIX 6.1 operating system. Multiparallel AD4 was compiled for BG/P with the XL C++ thread-safe cross-compiler v9.0 (bgxlC_r) and for POWER7 using the AIX XL C++ thread-safe v11.1 (xlC_r). For both POWER7 and BG/P, compiler optimization flag -O3 was used and the -qsmp = omp OpenMP option was specified, unless otherwise indicated. For POWER7 the -q64 flag was also used.

### Ligand Libraries and Parameters

The receptor-ligand complex 1HPV (indivinavir and HIV protease), and subsets of a diverse set of 34,841 compounds from the ZINC8 drug-like subset, were used to evaluate mpAD4 performance [22]. For the 1HPV, AutoDockTools (from MGLTools) was used to prepare the receptor and ligand [23]. Polar hydrogen atoms were added to the ligand and receptor .pdb files, and Gasteiger charges assigned. Indinavir libraries were then created with 4,000 copies (4 k indinavir), 8,000 copies (8 k indinavir), and 32,000 copies (32 k indinavir). The ZINC8 library ligands were prepared using the python scripts included in MGLTools package. To generate the 34,841 compound ZINC library (34 k ZINC), the 70% diversity subset of the *drug*-*like *subset was downloaded and compounds that failed any preparation step were discarded. A 9,000 compound subset of this library (9 k ZINC) was generated from the first 9,000 members of the 34 k ZINC library. To generate grid maps, grid box centers were defined as the center of the bound indinavir (1HPV), extending 60 grid points (0.375 Å per point) on each side. Unless otherwise specified, LGA runs were set at 20 (ga_runs), with population size (ga_popsize) of 150, energy evaluations (ga_num_evals) 250,000 and maximum number of generations (ga_num_generations) 27,000. All other parameter values were default for AutoDock 4.2. Except where indicated, the reuse_maps (gm = reuse) option was used in all benchmarks.

## Results and Discussion

**Figure 2:**
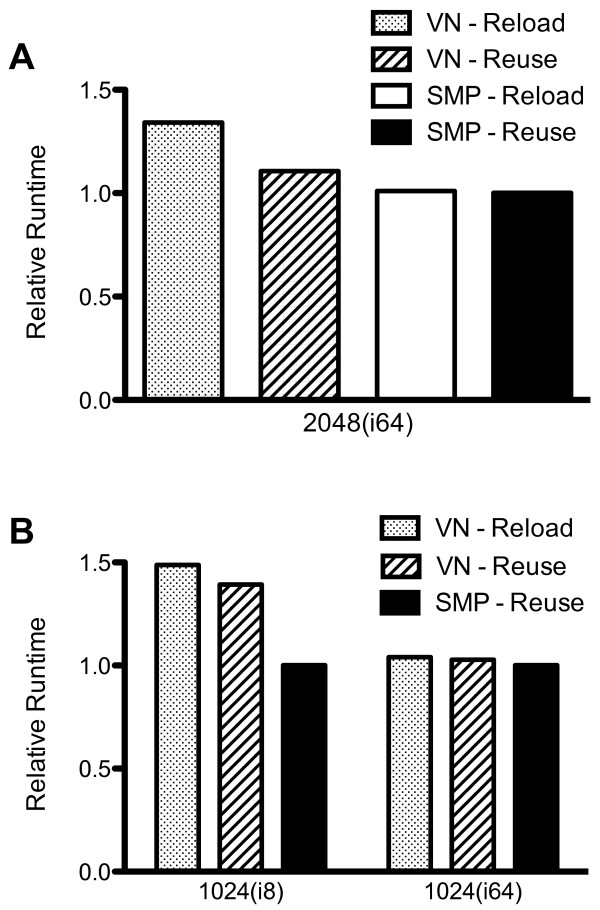
**Impact of grid map reuse, OpenMP multithreading, and I/O on mpAD4 execution speed**. (A) A 32,000 copy indinavir library was docked with mpAD4 on a 2,048(8,192) node(core) BG/P system in VN node mode (MPI + OpenMP with 1 OpenMP thread/node, 4× virtual nodes) with either grid map reloading or reuse and SMP mode (MPI + OpenMP with 4 OpenMP threads/node) with grid map reloading or reuse. The system was configured with 64 I/O nodes (1 I/O node per 128 cores). Runtimes were normalized to the fastest case (SMP, gm = reuse). (B) An 8,000 copy indinavir library was docked on a 1,024(4,096) node(core) BG/P system in VN mode (gm = reload or gm = reuse) and SMP mode (gm = reuse) configured with either 8 I/O nodes (I/O poor) or 64 I/O nodes (I/O replete). Runtimes were normalized to the fastest case (SMP, gm = reuse, I/O = 64).

To assess the performance characteristics of the hybrid parallelization and grid map reuse code, the 32 k indinavir library was docked on a 2,048(8,192) node(core) Blue Gene/P system with intermediate I/O settings (1 I/O node per 128 cores). Figure 2a shows the relative docking time in 4 different modes: VN(OMP = 1, gm = reload), VN(OMP = 1, gm = reuse), SMP(OMP = 4, gm = reload) and SMP(OMP = 4, gm = reuse). Grid map reuse reduced single-threaded execution time by approximately 17.5% due to reductions in I/O (Figure 2a). Multithreaded execution in SMP mode further reduced docking time by 10%, for an overall improvement of 25% over VN(OMP = 1, gm = reload) (Figure 2a). The improvement in docking speed observed with multithreading was due to system I/O bottlenecks experienced with single-threaded execution, as in VN mode each compute node CPU core receives an independent MPI task (8,192 in this instance), while in SMP mode only physical compute nodes (2,048 in this instance) receive a task, so that one fourth as many tasks run concurrently.

We examined the impact of system I/O on docking using I/O times recorded by the profiling code. The major sources of I/O when running mpAD4 were the loading of grid maps and the writing of log files, while MPI communication was a negligible percentage of network traffic. I/O saturation was most apparent at the end of the initial wave of docking jobs when multiple log files are simultaneously written to disk and grid maps for the next docking jobs are loaded. In our testing with the 32 k indinavir library, instituting grid map reuse diminished VN mode average file loading time by 77%, while SMP mode (OMP = 4, gm = reuse) file loading time was 1% that of VN mode (OMP = 1, gm = reload) (Table 1). Similarly, average log file writing time was reduced by 92% with grid map reuse, and over 99% in SMP mode (Table 1). Though grid map reuse significantly reduced I/O times in our tests, the impact on overall docking times was variable.

**Table 1:** Impact of grid map reuse and OpenMP multithreading on I/O.

	VN (gm = reload)				VN (gm = reuse)				SMP (gm = reuse)			
	**FLD**	**MAP**	**LIG**	**LOG**	**FLD**	**MAP**	**LIG**	**LOG**	**FLD**	**MAP**	**LIG**	**LOG**

ave	2.5	25.9	90.0	150.5	0.3	5.1	21.2	12.0	0.0	0.6	0.3	0.3
rms	8.0	50.8	335.8	584.1	1.5	13.8	40.8	55.4	0.1	2.4	0.3	0.3
med	0.3	13.4	1.9	0.2	0.0	0.0	1.5	0.3	0.0	0.0	0.2	0.2
max	247.2	542.3	3567.5	3630.0	54.6	187.9	469.6	546.8	1.7	14.0	2.9	3.2

%					14.6	19.7	23.6	8.0	1.1	2.5	0.4	0.3

A 32,000 copy indinavir library was docked with mpAD4 on a 2,048(8,192) node(core) BG/P system in VN node mode (MPI + OpenMP with 1 OpenMP thread/node, 4× virtual nodes) with either grid map reloading or reuse, and SMP mode (MPI + OpenMP with 4 OpenMP threads/node) with grid map reloading or reuse. Statistics for total duration (in seconds) of .fld map load time (FLD), atomic affinity map load time (MAP), ligand load time (LIG), and docking log write time (LOG) are shown for VN (gm = reload), VN (gm = reuse) and SMP (gm = reuse). Pairwise comparisons of average I/O times (seconds) between VN (gm = reload) and VN (gm = reuse) or SMP (gm = reuse) were calculated, and are shown as percent of VN (gm = reload) times. I/O differences between SMP (gm = reload) and (gm = reuse) were negligible, and are not shown.

### I/O and Performance

To further examine the contribution of I/O to the performance we observed, we docked an 8 k indinavir library on 1,024(4,096) node(core) BG/P system configured to be I/O poor (8 I/O nodes) or I/O replete (64 I/O nodes). In the I/O poor setting, VN mode with grid map reuse resulted in only a small increase in execution speed over VN(OMP = 1, gm = reload), while SMP mode (OMP = 4, gm = reuse) execution time was decreased by 33% (Figure 2b). Such differences were largely unapparent in an I/O replete configuration, where grid map reuse showed no benefit in overall docking speed, and SMP-mode gains were only 3% (Figure 2b). When I/O was sufficiently limited (e.g., 1,024(i8)), grid map reuse had limited impact on overall performance, likely because the I/O generated by the slave nodes writing log files was still sufficient to saturate the I/O poor system. Similarly, grid map reuse did not greatly improve performance in an I/O replete setting, as sufficient I/O capacity was available for simultaneous grid map loading and log file writing. Thus, grid map reuse greatly reduces I/O activity and can significantly improve docking performance in some settings, allowing larger systems to be effectively used for a given I/O capacity. Similarly, 4-way OpenMP multithreading reduces I/O by 75% for a given system size and I/O times by 90%, again allowing larger systems to be employed than with MPI alone.

### Hybrid Scalability

**Figure 3:**
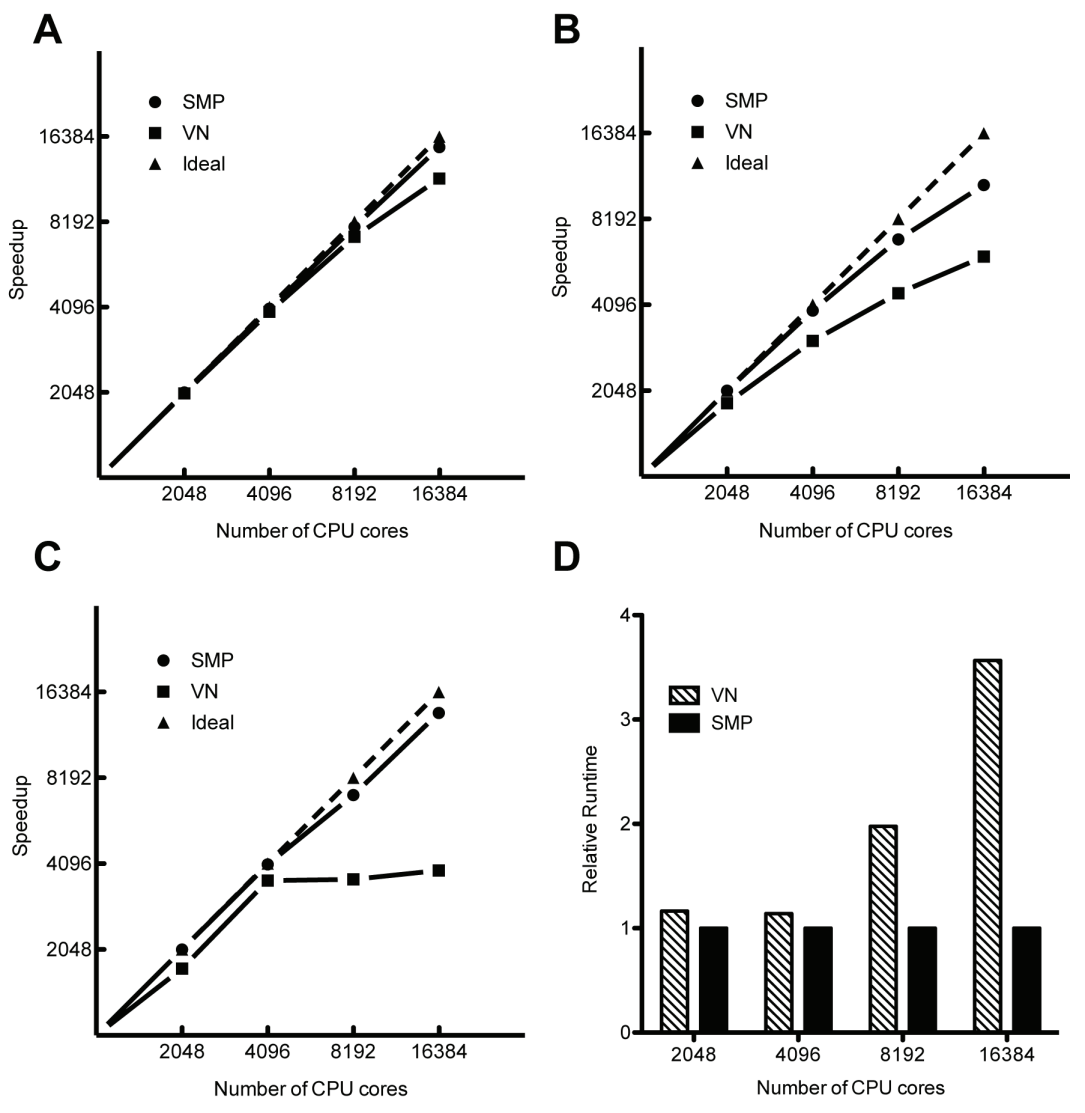
**Scaling of MPI alone versus MPI with OpenMP multithreading**. Virtual compound libraries were docked in VN and SMP modes on BG/P systems of 512(2,048), 1,024(4,096), 2,048(8,192) and 4,096(16,384) nodes(cores). (A) A 34,841 compound ZINC library was docked on variable BG/P systems sizes. Ideal speedup was calculated from the fastest 512 node result (SMP), and the relative speedup SMP and VN mode results were plotted. (B) A 9,000 compound subset ZINC library was docked on variable BG/P systems sizes, and ideal speedup was calculated from the fastest 512 node result (SMP). (C) A 4,000 copy indinavir library was docked on variable BG/P systems sizes, and an ideal speedup line was calculated from the fastest 512 node result (SMP). (D) Relative execution times for the 4,000 copy indinavir library were calculated for SMP and VN modes at each system size.

To test mpAD4 scalability, small molecule libraries including a 34 k ZINC, 9 k ZINC and 4 k indinavir were run on 512(2,048), 1,024(4,096), 2,048(8,192), and 4,096(16,384) node(core) BG/P systems. Figure 3a shows the speedup observed with the 34 k ZINC library in SMP and VN modes (gm = reuse). SMP mode scaling was nearly linear at approximately 92% ideal speed on the 16,384 core system. VN mode deviated to a greater degree at 72% ideal on the 16,384 core system, a 22% decrease from SMP-mode performance (Figure 3a). Interestingly, this performance decrease was not due to I/O differences, but instead reflected improved system utilization efficiency in the multithreaded execution mode. For both SMP and VN mode, deviations from ideal occur on larger systems as a virtual screen comes to the end and fewer ligands remain to be docked than capacity of the system, resulting in portions of the system remaining idle while the remaining active jobs finish. Multithreaded execution helps to alleviate this inefficiency in two ways: 1) when using multithreading there are fewer MPI nodes in the system and a virtual screen proceeds closer to completion before nodes become idle, and 2) individual dockings are executed more quickly when multithreaded, reducing time spent with idle nodes. For very large screening libraries, node utilization efficiency differences at the end of screening are unlikely to contribute to significant difference overall docking time. However, the opposite is true as library size shrinks in comparison to system size, as demonstrated in the scaling of the 9 K ZINC library where node utilization inefficiencies are apparent in both VN and SMP mode, though SMP mode is less effected (Figure 3b).

In addition to multithreading, node utilization can be improved by pre-ordering ligands to be docked by complexity (descending number of torsional angles). For a sorted 9 k indinavir library docked in VN mode on a 2,048 core system, sorting improved docking speed by 10%, though it was still 9% slower than SMP mode (data not shown). In cases where the availably of CPUs greatly exceeds the number of molecules to be screened, multithreading is particularly useful for increasing the usefully employable system size. For example, a 4 k indinavir library in single-threaded execution (VN, OMP = 1) is unable to take advantage of more than 4,000 cores (Figure 3c). In contrast, multithreading (SMP, OMP = 4) allows up to 16,000 cores to be employed, decreasing the docking time by over 70% (Figure 3c and Figure 3d). For larger systems, combining OpenMP multithreading with MPI allows for more efficient utilization of system resources at the end of screens. For smaller screens, multithreading has a clear advantages over serial execution when the number of available cores exceeds the number of ligand-receptor complexes to be docked.

### OpenMP Scalability

To test the scalability of the OpenMP implementation, a 1HPV complex was docked with mpAD4 on POWER7 p755 32-core server. Docking parameters were modified as follows: ga_runs = 128, ga_num_evals = 5,000,000. Table 2 shows apparent speedups and percent of ideal runtimes for a single docking of the 1HPV complex run as *serial *mpAD4 code (compiled without OpenMP) or utilizing from 1 to 32 OpenMP threads. On the POWER7 system, single-threaded OpenMP incurred a 12% overhead versus serial code. Overhead increased with thread number; with an apparent speedup of 1.5× for 2 threads to 22.3× for 32 threads (Table 2). Our testing on BG/P showed an overhead of approximately 10% for either OMP = 1 or OMP = 4, with little or no additional cost for 4 threads over 1 (data not shown). Due to the overhead incurred with OMP = 1 vs. serial, a user intending to use mpAD4 only in single-threaded applications may benefit from compiling mpAD4 without OpenMP. We anticipate the specific OpenMP overhead will vary with both system characteristics and compiler options. Though here we have only demonstrated multithreading up to 32 cores, the code is currently implemented to allow up to 128 simultaneous threads, which we expect will allow further improvements total in docking speed.

**Table 2:** OpenMP Multithreading Speedup

Threads	Speedup	% Ideal
serial	1.0×	100
1	0.9×	88
2	1.5×	75
4	3.0×	74
8	5.8×	72
16	11.3×	71
32	22.3×	70

The speedup using OpenMP threads was calculated for 1 to 32 threads on a 3.3 GHz POWER7 p755 32-core server. A reference speed was calculated using mpAD4 compiled without OpenMP (serial). The numbers are scaled to a single serial docking run with the default benchmark parameters with modifications: ga_runs = 128, ga_num_evals = 5,000,000.

### Output Comparison

The binding modes generated by single or multithreaded execution of mpAD4 were determined for a set of 76 crystallographically determined ligand-protein complexes using a BG/P system size of 512 cores in SMP(OMP = 4) and VN(OMP = 1 or serial) modes [24]. For each docked complex, pairwise RMSDs were calculated for the overall lowest energy ligand and lowest energy member of largest ligand cluster. When the lowest energy ligand was not also a member of the largest ligand cluster, the lesser pairwise RMSD value was used. The RMSDs from VN(serial) to VN(OMP = 1) or SMP(OMP = 4), and between VN(OMP = 1) and SMP(OMP = 4) were calculated (Table 3). The mean RMSD values in all three comparisons were less than 1.0, with median values less than 0.2 (Table 3). We therefore consider the outputs to be substantially similar.

**Table 3:** Parallel and Serial Docking of 76 Receptor-Ligand complexes

RMSD Comparisons			
	**s vs 1**	**s vs 4**	**1 vs 4**

ave	0.414	0.439	0.366
rms	0.525	0.667	0.510
med	0.168	0.170	0.139
min	0.005	0.008	0.003
max	2.526	4.332	3.255

76 Receptor-Ligand complexes were docked using a 128 node (512 core) BG/P system using the benchmark parameters with modifications: ga_runs = 70, ga_num_evals = 5,000,000. Pairwise RMSD values between VN (serial) and VN (OMP = 1), VN (serial) and SMP (OMP = 4), and VN (OMP = 1) and SMP (OMP = 4) for both the lowest energy docked ligands and the lowest energy member of the largest ligand cluster were calculated. When the pairwise RMSD between lowest energy-lowest energy and largest cluster-largest cluster were not equal, the lesser RMSD value was used (approximately 20% of comparisons). Statistics for the three comparisons were calculated.

### Performance Expectations

The implementation of MPI and OpenMP in mpAD4 is portable to systems with a suitable compiler and the required libraries. In the case of distributed-memory architectures using either Intel or AMD ×86 microprocessors, we expect similar trends in terms of performance. Environmental factors that may have a large impact on performance are network bandwidth, compute node microprocessor speed, memory and the availability of node local disk storage (potentially ameliorating I/O issues associated with writing log files). Multiparallel AD4 generates little MPI communication, and we therefore anticipate that it will scale well even on clusters with limited I/O bandwidth if they possess node local disk storage and sufficient RAM to store grid maps in memory. Similarly, we would predict that the OpenMP multithreading will generate performance gains on any modern multicore microprocessor, though overhead and absolute scalability may vary with compilers, compiler options and microprocessor architecture.

## Conclusions

We have parallelized AutoDock 4.2 using MPI and OpenMP to create mpAD4, a standards compliant and portable parallel implementation of AutoDock, with user customizability in the balance between serial and parallel execution, a capability to reuse grid maps, and extensive profiling features for performance monitoring. In our tests, grid maps reuse drastically reduced system I/O, allowing for nearly linear scaling of mpAD4 on system sizes of up to 16,384 CPU cores. OpenMP multithreading scaled up to 32 threads, resulting in a maximum speedup of 22× over single-threaded execution. We propose three potential use cases for mpAD4: 1) combining MPI and OpenMP parallelization on large systems to balance system-level and node-level parallelization to manage I/O and achieve the best possible throughput, 2) enabling larger systems to be used for screening small libraries, and to improve system utilization at all library sizes, 3) facilitating the rapid docking of one or a small number of ligand-recepor complexes on shared memory systems.

## Availability and Requirements

Project name: mpAutoDock 4.2

Project home page: http://autodock.scripps.edu/downloads/multilevel-parallel-autodock4.2

Operating system(s): Platform independent

Programming language: C++

Other requirements: MPI (MPICH2), OpenMP

License: GNU GPL v3

## Competing interests

The authors declare that they have no competing interests.

## Authors' contributions

APN participated in the design of this work, performed validation and benchmarking of the parallel code, and wrote this manuscript. PKC parallelized the AutoDock code, and assisted in drafting this manuscript. JPK participated in the design of this work, and in revising this manuscript for publication. DJK assisted in the analysis of the data, and in revising this manuscript for publication. CPS conceived this study, participated in the design of this work, coordinated its execution, and helped to revise this manuscript for publication. All authors read and approved the final manuscript.
